# Comparison of western diet-induced obesity and streptozotocin mouse models: insights into energy balance, somatosensory dysfunction, and cardiac autonomic neuropathy

**DOI:** 10.3389/fphys.2023.1238120

**Published:** 2023-10-11

**Authors:** Nadia Elshareif, Emily Gornick, Chaitanya K. Gavini, Gregory Aubert, Virginie Mansuy-Aubert

**Affiliations:** ^1^ Department of Cell and Molecular Physiology, Stritch School of Medicine, Loyola University Chicago, Maywood, IL, United States; ^2^ Department of Biomedical Sciences, University of Lausanne, Lausanne, Switzerland; ^3^ Division of Cardiology, Department of Internal Medicine, Stritch School of Medicine, Loyola University Chicago, Maywood, IL, United States; ^4^ Clinical Development, CSL Vifor, Glattbrugg, Switzerland

**Keywords:** streptozotocin, obesity, neuropathy, energy expenditure, insulin resistance, cardiac dysfunction

## Abstract

Metabolic disorders such as obesity and type 2 diabetes (T2D) are increasingly prevalent worldwide, necessitating a deeper comprehension of their underlying mechanisms. However, translating findings from animal research to human patients remains challenging. This study aimed to investigate the long-term effects of Streptozotocin (STZ) on metabolic, cardiac, and somatosensory function in mice fed a Western diet (WD) of high fat, sucrose, and cholesterol with low doses of STZ administration compared to mice fed WD alone. In our research, we thoroughly characterized energy balance and glucose homeostasis, as well as allodynia and cardiac function, all of which have been previously shown to be altered by WD feeding. Notably, our findings revealed that the treatment of WD-fed mice with STZ exacerbated dysfunction in glucose homeostasis via reduced insulin secretion in addition to impaired peripheral insulin signaling. Furthermore, both WD and WD + STZ mice exhibited the same degree of cardiac autonomic neuropathy, such as reduced heart rate variability and decreased protein levels of cardiac autonomic markers. Furthermore, both groups developed the same symptoms of neuropathic pain, accompanied by elevated levels of activating transcription factor 3 *(Atf3)* in the dorsal root ganglia. These discoveries enhance our understanding of metabolic activity, insulin resistance, neuropathy, and cardiac dysfunction of diet-induced models of obesity and diabetes. The exacerbation of impaired insulin signaling pathways by STZ did not lead to or worsen cardiac and somatosensory dysfunction. Additionally, they offer valuable insights into suitable diet induced translational mouse models, thereby advancing the development of potential interventions for associated conditions.

## Introduction

Metabolic disorders, including obesity, and type 2 diabetes (T2D) are witnessing a rapid surge worldwide, primarily driven by the complex interplay of unhealthy dietary patterns and sedentary lifestyles. This escalating trend poses a substantial burden on healthcare systems globally, necessitating urgent attention and comprehensive strategies to address associated complications (Leung et al., 2017; Regufe et al., 2020). Understanding the intricate mechanisms behind metabolic disorders and their associated complications, including cardiovascular disease, cancer, and various forms of neuropathy, is of paramount importance. Achieving this requires bridging the translational gap between rodent models and human patients, calling for thorough testing and comparison of multiple species and models.

While genetic models such as the *ob/ob* and *db/db* mouse models have proven instrumental in unraveling the underlying mechanisms of T2D, it is noteworthy that mutations in leptin and leptin receptors, which these models represent, are rarely observed in human patients (Daniels Gatward et al., 2021). Therefore, exploring alternative avenues becomes imperative to gain a comprehensive understanding of metabolic disorders in humans (O'Brien et al., 2014; Drel et al., 2006; Sullivan et al., 2007). Such genetic models may not capture the impact of metabolic dysregulation on targeted tissue such as heart, peripheral neurons and their dysfunction in various physiological systems (Da Costa Goncalves et al., 2009). In addition, the gradual progression of disease is challenging to reproduce as they develop diabetic phenotypes at as little as 4 weeks old, with death that can occur in as little of 24 weeks of age (Biessels et al., 2014).

Many findings on obesity and related metabolic complications were generated using special diets, including a high amount of fat (between 60% and 70%) known as the high-fat diet (HFD), high sucrose diet (HSD), and Western diet (WD) which comprehensively mimics human western diet as it is comprised of high fat, sucrose and cholesterol (O'Brien et al., 2014; Gilbert et al., 2011; Sumiyoshi et al., 2006). These diets have been used to understand both central metabolic dysfunction and associated comorbidities of obesity and early diabetes. While rodents develop early signs of glucose intolerance and insulin resistance, not all classical features of T2D are achieved via diet alone.

It is true that the glucose analog streptozotocin (STZ) has been widely used at high doses to model type 1 diabetes in rodents due to its cytotoxic effects to pancreatic beta cells (Nahdi et al., 2017). Newer studies have adapted protocols to generate a T2D model using low doses (50–75 mg/kg) of STZ in HFD-fed mice with the goal of providing a mouse model that emulates the human state of disease and its progression from obesity and prediabetes to later stages of T2D in adulthood (Cerf, 2013; Carper et al., 2020; Daniels Gatward et al., 2021). This paradigm involves STZ injection in obese mice at as little as 7 weeks of HFD feeding, where mice develop robust glucose intolerance and insulin resistance phenotypes compared to obese mice given HFD alone (Leung et al., 2017; Nahdi et al., 2017; Daniels Gatward et al., 2021). These paradigms note both glucose intolerance and insulin resistance at 24 weeks in HFD-STZ mice, where no differences in measures of peripheral neuropathy phenotypes measured at 16, 24, and 36 weeks of the study between HFD and HFD-STZ groups (Leung et al., 2017; Nahdi et al., 2017; Daniels Gatward et al., 2021). A comprehensive characterization of this model remains incomplete, emphasizing the need to longitudinally compare the metabolic phenotype of diet-induced obese mice with that of obese mice treated with STZ, especially with the incorporation of WD which has not been evaluated with STZ injection. We used WD to induce early adult obesity and injected at 7 weeks to mature adult mice. We believe this model mimics childhood/early adult obesity, glucose intolerance and progression to T2D at the mature adult age.

In summary, we investigate the longitudinal effects of WD feeding alone versus the additional use of low doses of STZ in WD-fed mice on metabolic, neuronal, and cardiac dysfunction in order to provide data that will help preclinical research to identify appropriate models to study metabolic dysfunction and associated comorbidities (Furman, 2021; Huo et al., 2021).

## Results

### Low doses of STZ in WD-fed mice reduces serum insulin secretion and impairs peripheral insulin signaling

**FIGURE 1:**
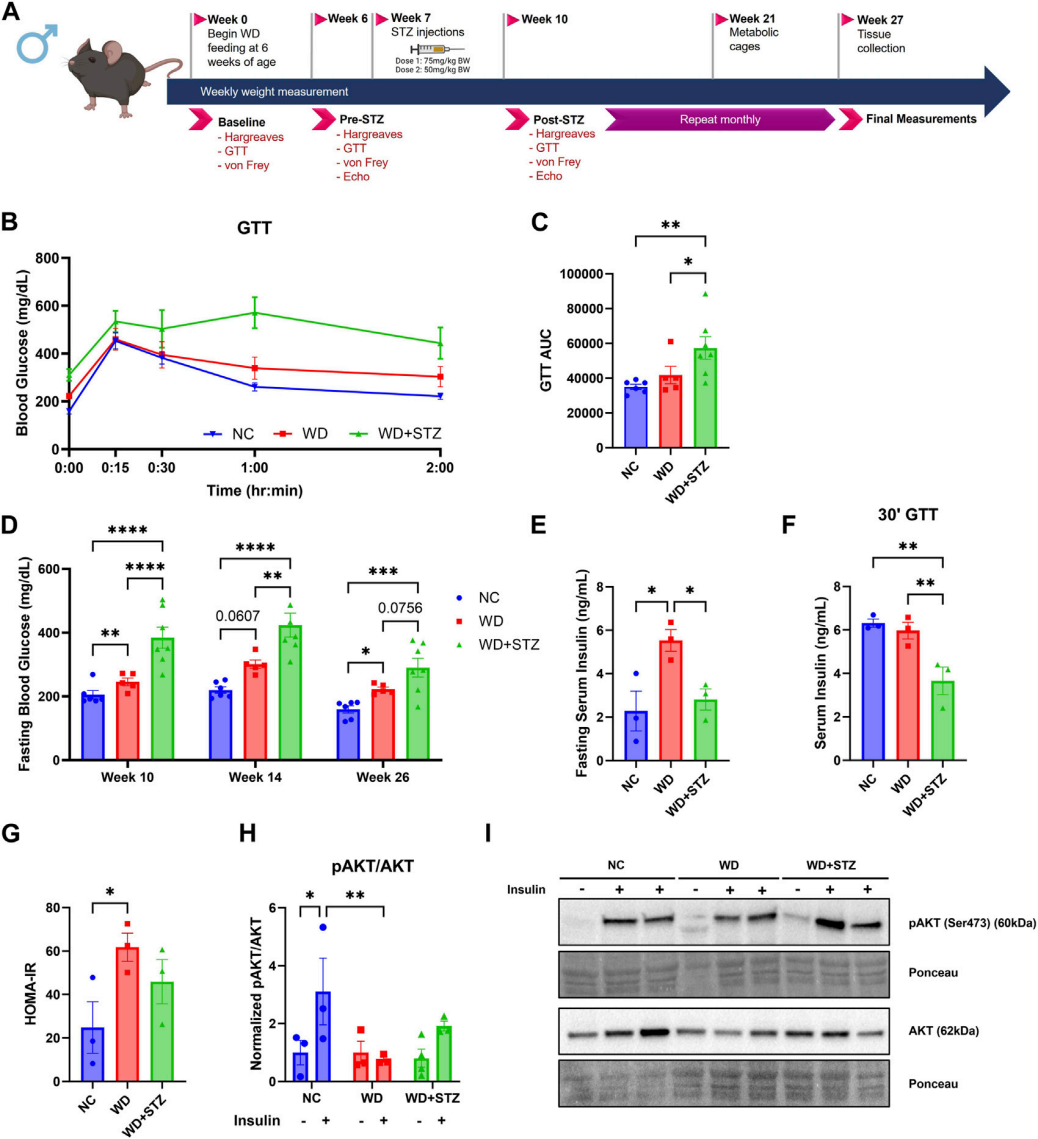
Low doses of STZ in WD-fed mice reduces serum insulin secretion and impairs peripheral insulin signaling. **(A)** Experimental paradigm where 6-week-old mice were given normal chow (NC) or western diet (WD) for 26 weeks. At week 7, mice were injected with multiple doses of streptozotocin (STZ) or vehicle. **(B)** Week 26 glucose tolerance test (GTT) and **(C)** area under the curve (AUC). **(D)** Blood glucose measurements after 4 h fast. **(E)** Week 26 fasting serum insulin levels, **(F)** serum insulin levels 30 min post-glucose injection during GTT, and **(G)** calculated Homeostatic Model Assessment for Insulin Resistance (HOMA-IR) scores (*n* = 3/group). **(H)** The ratio of pAKT to AKT protein levels from soleus muscle with **(I)** representative immunoblot images, where mice were given intraperitoneal injections of either insulin (.75 U/kg bodyweight) or vehicle before euthanization at week 26 (*n* = 3–4/group) (two-way ANOVA). Statistical analyses were done using one-way ANOVA unless reported otherwise. Data are mean ± SEM (*p < 0.05, **p < 0.01, ***p < 0.005, ****p < 0.0001).

The efficacy of low doses of STZ in modeling T2D in WD-fed mice is not well understood, thus we aimed to compare the glucose phenotype of WD and WD + STZ mice. The mice were fed with WD for 26 weeks and received two injections of STZ (75 and 50 mg/kg body weight) or vehicle 3 days apart after 7 weeks of WD feeding (Figure 1A). After 26 weeks of WD feeding, WD + STZ mice shown significantly higher glucose levels during the glucose tolerance test (GTT) than WD- and NC-fed mice (Figures 1B, C). Throughout the experiment, WD + STZ mice consistently displayed higher fasting blood glucose levels after STZ injection compared to NC-fed mice, which was sustained until the end of the study (Figure 1D). As expected, WD-fed mice also demonstrated higher fasting blood glucose levels compared to NC-fed mice, as previously reported (Figure 1D) (Gavini et al., 2018; Elshareif et al., 2022). At week 14, WD + STZ mice had higher fasting glucose levels than WD-fed mice, and by week 26, fasting glucose levels were trending higher than those in WD-fed mice (Figure 1D).

To investigate if the observed differences between the WD and WD + STZ groups were associated with changes in circulating insulin levels, serum insulin was measured after a 4-h fast. After 26 weeks, WD + STZ mice shown significantly lower fasting serum insulin levels compared to WD-fed mice, with a level of 2.8 ng/mL (Figure 1E). This level was comparable to the insulin levels in the NC group at 2.3 ng/mL, while WD-fed mice displayed the highest circulating concentration of 5.5 ng/mL post-fast (Figure 1E). Furthermore, during the glucose challenge at week 26, where tail blood samples were obtained 30 min post-glucose injection, WD + STZ mice demonstrated lower serum insulin levels compared to NC and WD-fed mice (Figure 1F). This suggested that the two paradigms model different stages of T2D, with WD representing prediabetes with pronounced peripheral insulin resistance and WD + STZ representing later stages of T2D with the additional characteristic of impaired insulin secretion. This hypothesis was confirmed using calculated Homeostatic Model Assessment for Insulin Resistance (HOMA-IR) scores. The WD-fed mice had the highest average index score of 60, significantly higher than the average NC score of 25 (Figure 1G). The WD + STZ mice had an average index score of 45, indicating an intermediate value between WD-fed and NC-fed mice (Figure 1G). Our findings indicate that WD and WD + STZ mice exhibit peripheral insulin resistance based on the HOMA-IR scores compared to NC group.

To further confirm this observation, we conducted *in vivo* testing to assess the response to insulin in muscle in the control, WD, and WD + STZ groups. Insulin signaling was evaluated by immunoblotting for the Ser473 residue of phosphorylated protein kinase B (pAKT) and total AKT in the soleus muscle after insulin injection (0.75 U/kg body weight). Consistent with expectations, NC-fed mice injected with insulin demonstrated an increase in the pAKT/AKT ratio compared to the vehicle (Figures 1H, I). In contrast, WD-fed mice and WD + STZ mice did not show a significant change in the protein ratio of pAKT/AKT when injected with insulin compared to the vehicle (Figures 1H, I). Moreover, there was a significant difference in ratios between insulin-injected WD-fed mice and NC-fed mice (Figures 1H, I), while no significant difference was observed between the NC and WD + STZ insulin groups (Figures 1H, I).

These findings suggest that WD mice exhibit insulin resistance while maintaining normal insulin secretion, a more characteristic model of earlier stages of T2D. On the other hand, the combined use of STZ and WD appears to produce a more complex diabetic phenotype characterized by a combination of impaired insulin secretion and peripheral insulin resistance.

### WD and WD + STZ mice display similar features of early cardiac dysfunction concomitant with a decrease in autonomic innervation

**FIGURE 2:**
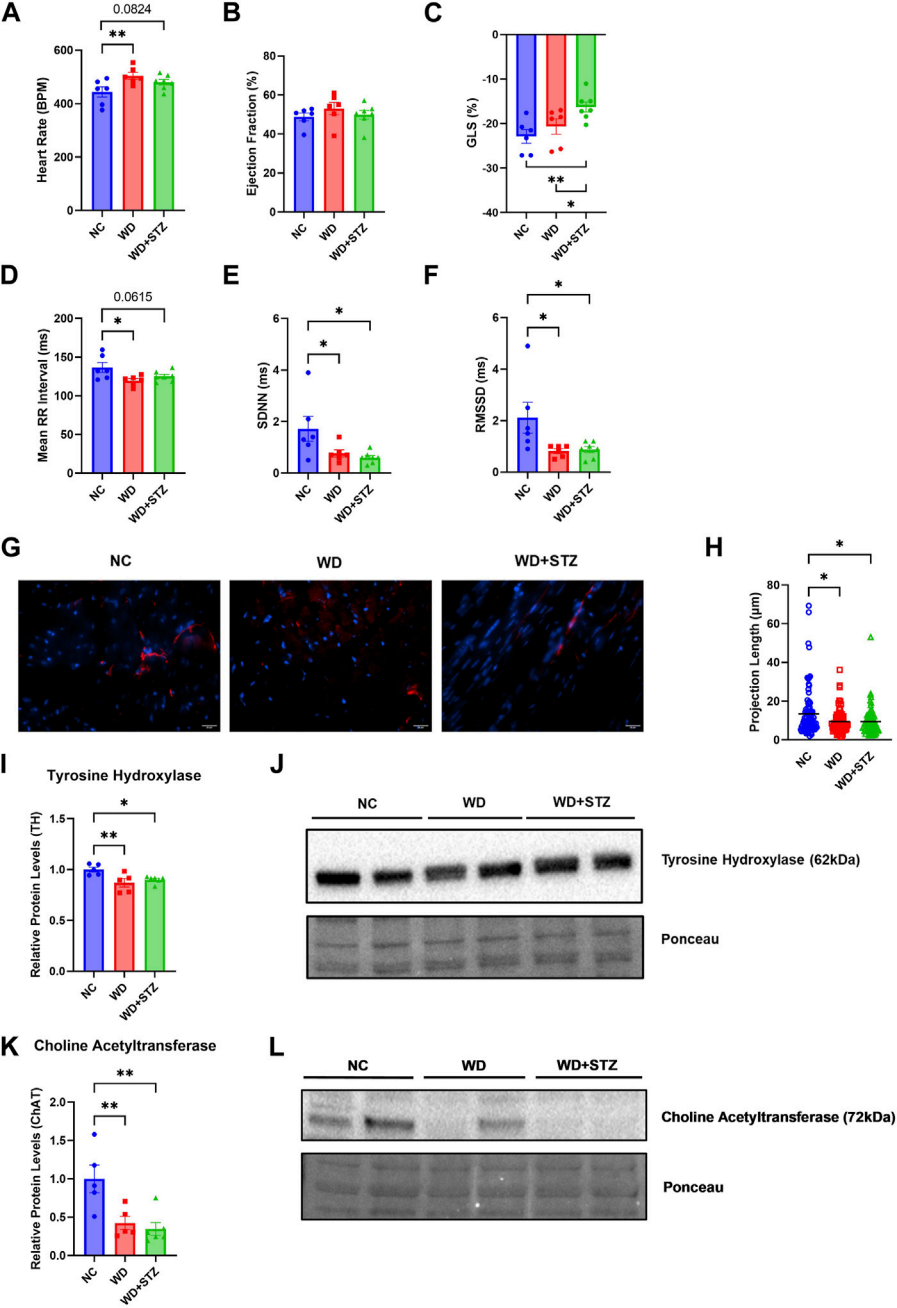
WD and WD + STZ mice display features of early cardiac dysfunction concomitant with a decrease in autonomic innervation. Week 26 echocardiography measurements of **(A)** heart rate, **(B)** ejection fraction, and **(C)** global longitudinal strain (GLS). **(D)** Heart rate variability assessment of mean RR interval, **(E)** standard deviation of NN intervals (SDNN), and **(F)** root mean square of successive differences between normal heartbeats (RMSSD). **(G)** Representative images of tyrosine hydroxylase (TH)+ cardiac fibers (TH-red, nucleus-DAPI/blue) and **(H)** calculated TH+ fiber projection length. **(I)** Relative TH protein levels compared to total protein staining with **(J)** representative immunoblot images. **(K)** Relative Choline Acetyltransferase (ChAT) levels compared to total protein staining with **(L)** representative immunoblot images. Statistical analyses were done using one-way ANOVA (*n* = 6–7/group). Data are mean ± SEM (*p < 0.05, **p < 0.01, ****p < 0.0001).

Throughout the paradigm, we performed echocardiograms on the mice to measure indices of cardiac dysfunction between WD-fed mice and WD + STZ mice. At the end of the paradigm, WD-fed mice had higher heart rates, with an average of 505 beats per minute (BPM), compared to the average NC heart rate of 445 BPM (Figure 2A). The WD + STZ mice had trending higher heart rates than the NC-fed mice at an average of 480 BPM (Figure 2A). There was no evident change in ejection fraction between groups (Figure 2B). This was clear throughout the paradigm (Supplementary Figure S1A). However, the average global longitudinal strain (GLS) in WD + STZ mice was −16%, which was significantly reduced compared to NC and WD-fed mice (Figure 2C). This altered GLS was evident at week 10 compared to that in WD-fed mice and at week 14 compared to both WD- and NC-fed mice (Supplementary Figure S1B). No changes in left ventricle posterior wall diameter (LVPWd) were detected in WD and WD + STZ groups compared to NC-fed mice (Supplementary Figure S1C).

Given that both the WD and WD + STZ groups had characteristics of early cardiac dysfunction, we were interested in whether this phenotype was accompanied by changes in cardiac autonomic tone that can manifest in prediabetic and obese humans (Williams et al., 2019). Using the echocardiogram data, we determined whether the WD and WD + STZ groups developed changes in heart rate variability (HRV), a common indicator in cardiac autonomic neuropathy (Yadav et al., 2017; Williams et al., 2019). The mean RR interval, standard deviation of normal RR intervals (SDNN), and root mean square of successive differences between normal heartbeats (RMSSD) were calculated. At the end of the paradigm, the average mean RR interval was 120 and 125 ms for WD and WD + STZ groups, respectively, compared to the NC mean RR interval of 137 ms (Figure 2D). The average SDNN was 0.77 and 0.6 ms for WD and WD + STZ groups respectively, which was significantly lower than the NC SDNN average of 1.71 ms (Figure 2E). This observation was comparable to what we saw with the RMSSD, which was 0.81 ms for WD and 0.87 ms for WD + STZ mice compared to 2.11 ms average from the NC-fed mice (Figure 2F).

We hypothesized that the features of cardiac dysfunction and decreases in HRV indices may be associated with loss of autonomic innervation. To determine whether there was a loss in cardiac sympathetic tone, we stained perfused heart tissue with tyrosine hydroxylase (TH), a rate limiting enzyme for catecholamine synthesis (Figure 2G) (Qin et al., 2021). We quantified the projection length of TH + fibers, where both WD and WD + STZ cardiac tissues had the same decrease in projection length compared to NC (Figure 2H). Immunoblots for cardiac TH levels yielded comparable results, where WD and WD + STZ groups had an average of 10% and 15% lower protein levels, respectively, compared to NC (Figures 2I, J). We also observed a decrease in protein levels for the parasympathetic marker choline acetyltransferase (CHAT), from cardiac tissue of both WD and WD + STZ groups (Figures 2K, L) (Qin et al., 2021). These data suggest that both models display the same characteristics in heart dysfunction potentially due to cardiac autonomic neuropathy.

### WD and WD + STZ mice develop somatosensory dysfunction

In the context of diabetic neuropathy-associated somatosensory dysfunction, we conducted a longitudinal comparison of mechanical and thermal sensitivity behaviors in mice subjected to a WD or a combination of WD and STZ (WD + STZ). Conversely, both WD and WD + STZ mice consistently exhibited lower mechanical allodynia thresholds compared to NC mice throughout the entire paradigm (Supplementary Figure S2A). At weeks 10 and 14 of the paradigm, both WD and WD + STZ mice exhibited lower thermal latency than NC-fed mice (Supplementary Figure S2B). However, by the end of the paradigm, neither the WD nor WD + STZ mice demonstrated significant thermal hyperalgesia.

To investigate whether this observed hypersensitivity phenotype was accompanied by neuronal injury in the dorsal root ganglia (DRG), potentially resulting from the effects of the WD and STZ, we performed quantitative polymerase chain reaction (qPCR) targeting the activating transcription factor 3 (*Atf3*) mRNA levels in the lumbar DRG, as previously described (Nascimento et al., 2011). Remarkably, we found that both WD and WD + STZ mice exhibited a similar increase in *Atf3* mRNA levels, ranging between 20% and 30% higher than those observed in NC DRG (Supplementary Figure S2C). This data shows that STZ treatment did not worsen the neuropathy condition.

### WD and WD + STZ mice have similar body composition and energy expenditure profiles

**FIGURE 3:**
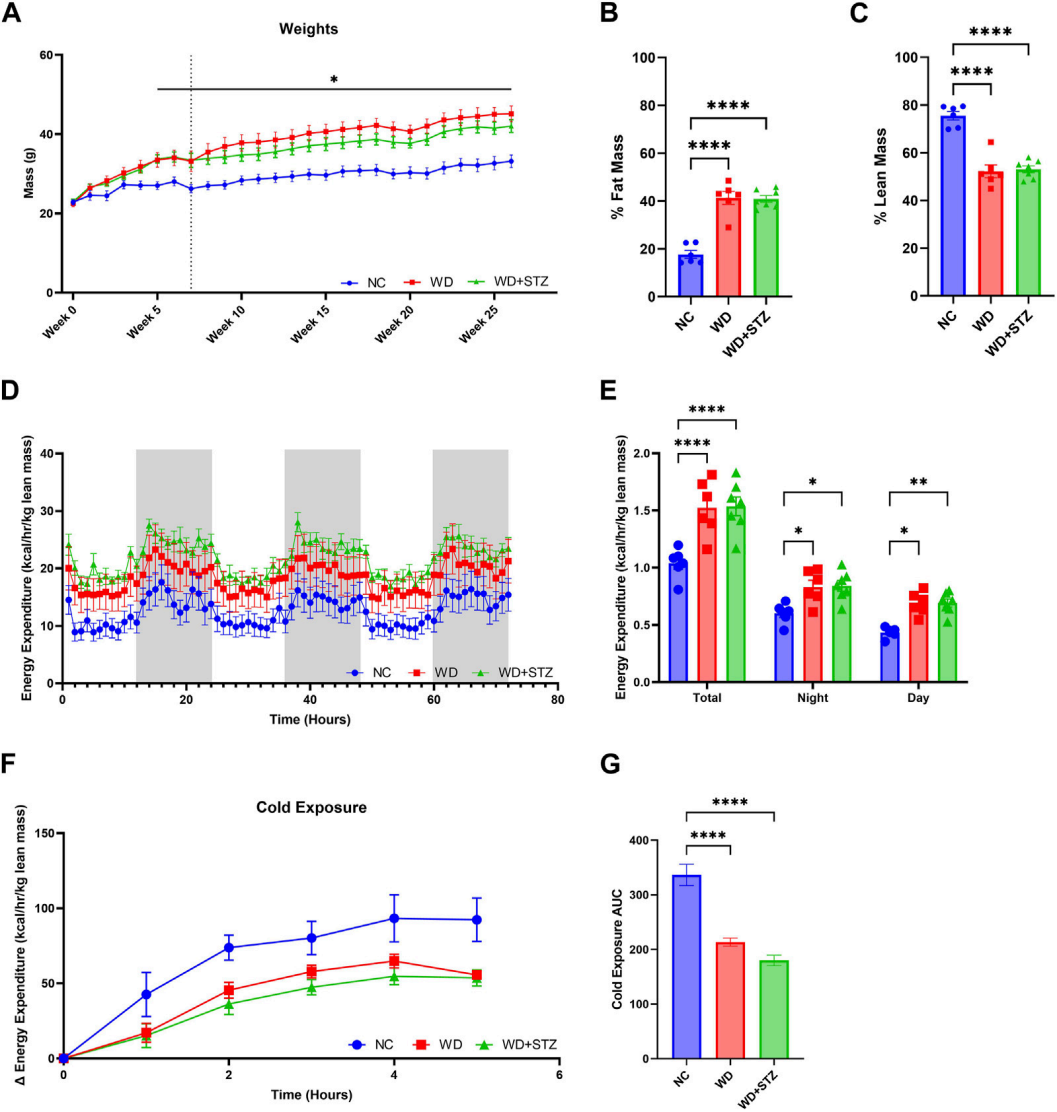
WD and WD + STZ mice have similar body composition and energy expenditure profiles. **(A)** Weekly weight measurements where week 0 is the start of diet feeding (Dotted line indicates injection at week 7, Repeated measures two-way ANOVA, *p < 0.05 WD/WD + STZ vs. NC). **(B)** Body composition analysis of fat mass and **(C)** lean mass at week 21 of the paradigm. **(D)** Hourly and **(E)** cumulative energy expenditure profiles over 72 h. **(F)** Change in energy expenditure during 4-h cold exposure at 4°C, and **(G)** area under the curve (AUC). Statistical analyses were done using one-way ANOVA unless reported otherwise (*n* = 6–7/group). Data are mean ± SEM (*p < 0.05, **p < 0.01, ****p < 0.0001).

We sought to compare further the metabolic phenotype of WD-fed mice together with WD-fed mice treated with STZ. After 5 weeks of feeding, WD and WD + STZ mice were significantly heavier than their NC counterparts (Figure 3A). The mass of WD and WD + STZ mice comprised approximately 40% fat mass (Figure 3B) and 50% lean mass (Figure 3C) compared to the NC-fed mice composed of 20% fat mass (Figure 3B) and 80% lean mass (Figure 3C).

We then sought to determine whether STZ further impaired the metabolic energy expenditure profile of WD-fed mice. At week 21 of the paradigm, mice were subjected to indirect calorimetry for 72 h (Figure 3D). Both the WD and WD + STZ mice displayed elevated rates of energy expenditure during both the day and night, resulting in a 25% increase compared to the NC-fed mice (Figure 3E). To assess the mice’s ability to regulate their body temperature by modulating energy expenditure, we subjected them to a 72-h indirect calorimetry at 24.5°C, followed by a 4-h cold challenge (Figure 3F). While both the WD and WD + STZ mice exhibited higher energy expenditure at ambient temperature compared to the NC group, their response to cold exposure was blunted (Figures 3F, G).

In summary, both the WD and WD + STZ groups maintained similar body composition and energy expenditure profiles at both normal temperatures and during the cold challenge.

### WD and WD + STZ mice have similar respiratory exchange ratios but different feeding behaviors

**FIGURE 4:**
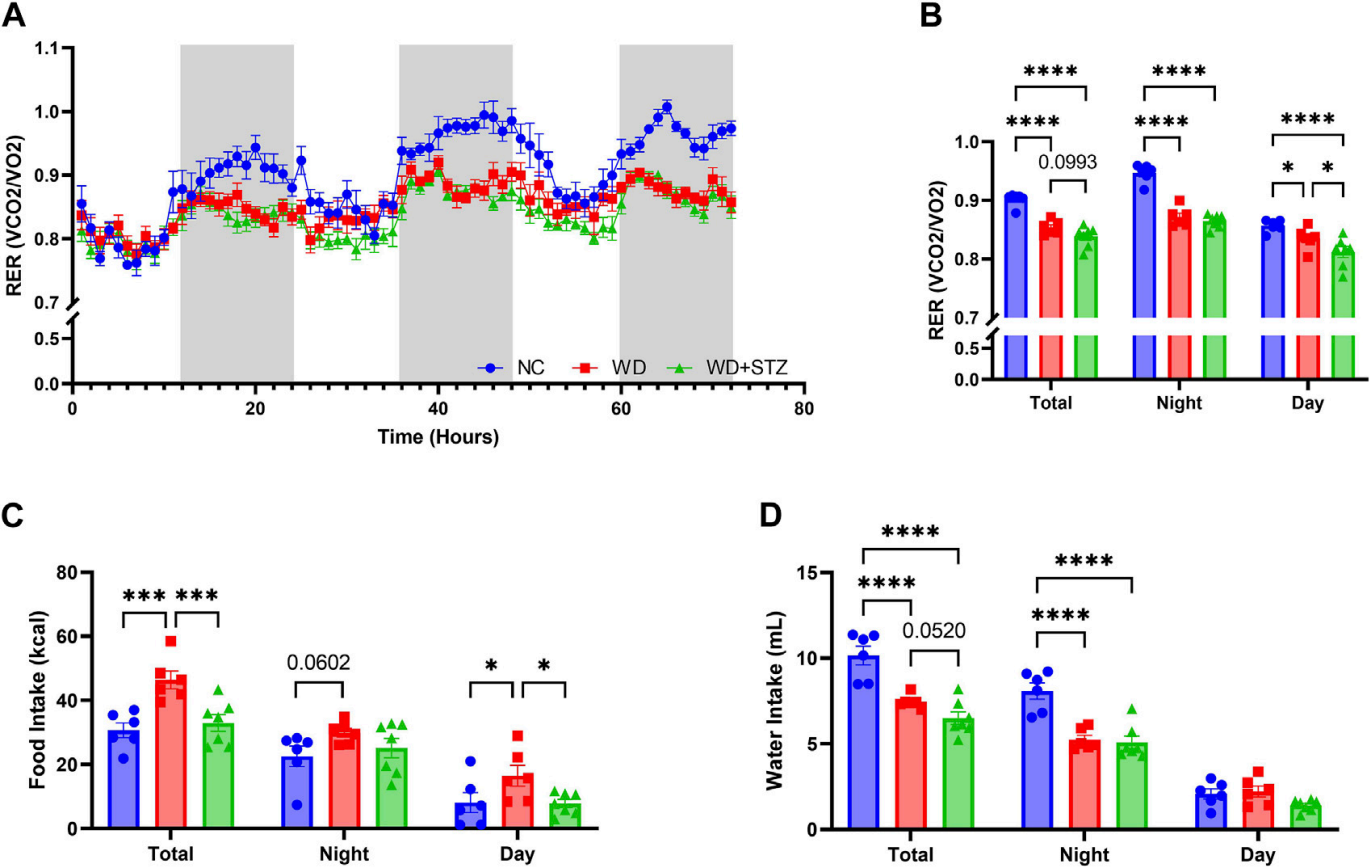
WD and WD + STZ mice have similar respiratory exchange ratios but different feeding behaviors. **(A)** Hourly and **(B)** cumulative respiratory exchange ratio (RER) measured during indirect calorimetry. **(C)** Cumulative food intake and **(D)** water intake during indirect calorimetry. Statistical analyses were done using one-way ANOVA (*n* = 6–7/group). Data are mean ± SEM (*p < 0.05, ***p < 0.005, ****p < 0.0001).

During indirect calorimetry experiments, we calculated the respiratory exchange ratio (RER) to determine the substrate type utilized by mice for energy production (Figure 4A). On average, the RER for NC-fed was 0.90, whereas the RER was lower for mice on a WD and WD + STZ at 0.85 and 0.84, respectively (Figure 4B). The RER of NC mice was highest during nighttime, averaging at 0.95, while the WD and WD + STZ mice maintained significantly lower ratios of 0.85 (Figure 4B). During the daytime, all groups exhibited the lowest RER values, with NC-fed mice recording 0.86 (Figure 4B). Both WD and WD + STZ mice had significantly lower RER values than NC, with rates of 0.84 and 0.81, respectively (Figure 4B). These findings indicate that WD and WD + STZ mice primarily utilize fat as a fuel source, whereas NC-fed mice predominantly rely on carbohydrates, especially during nighttime when they are most active. Notably, despite having similar body weights, food intake analysis revealed that the WD group consumed significantly more food than the WD + STZ and NC groups overall, with higher daytime food intake (Figure 4C). Furthermore, both the WD and WD + STZ groups exhibited lower water intake compared to the NC group, particularly during nighttime (Figure 4D).

## Discussion

The present study aimed to comprehensively characterize the complex interplay between metabolic dysfunction and neuronal complications, encompassing various physiological systems. First, we use a WD and not HFD to induce a phenotype that can be translated to an obese metabolic syndrome. We conducted a 26-week study using low-dose STZ injections in obese mice, which has previously been shown to intensify glucose intolerance and insulin resistance phenotypes at as little as 16 weeks post-injection without producing a type I diabetes phenotype (O'Brien et al., 2018; Yorek et al., 2015). STZ is a glucose analog that is known to damage tissues expressing high levels of glucose transporter 2 (GLUT2), particularly in insulin-secreting pancreatic beta cells (Szkudelski, 2001; Nahdi et al., 2017). WD feeding alone possesses a gradual impact on peripheral insulin resistance through nutrient excess, and prolonged consumption disrupts insulin secretion as a result of sustained beta cell hyperexcitability (Cerf, 2013). STZ Injections were performed at 7 weeks of WD feeding to follow a translatable disease progression timeline. Indeed, we wanted to create a model of obesity that are insulin and glucose intolerant that progress to type II diabetes in the early adult age to evaluate early changes and early events responsible of obesity complications (O'Brien et al., 2018; Yorek et al., 2015).

We have previously published that WD-fed mice developed glucose intolerance and insulin resistance after 11 weeks of feeding (Elshareif et al., 2022). Building upon these findings and of those who utilize HFD-STZ mice, our current study compared glucose homeostasis between WD + STZ mice and mice fed WD alone. We observed that WD + STZ mice exhibited glucose intolerance and impaired insulin secretion during fasting and post-glucose challenge, which are key features of T2D. As documented in previous studies with the HFD-STZ mouse model, we aimed to recapitulate the results of their GTT, where 4-h fasting was performed (O'Brien et al., 2018). Given the number of experiments at each time points, longer fasting times may introduce confounding metabolic stressors including changes in catabolic gene expression and weight loss which is time-dependent with longer fasting periods (Ayala et al., 2010; Carper et al., 2020). This may explain the lack of significant difference in GTT results between NC- and WD-fed mice and longer fasting periods may present more robust differences in GTTs performed on this model and may be optimal for future studies. WD-fed mice had a higher likelihood of insulin resistance, as indicated by HOMA-IR scores considering fasting insulin levels. Interestingly, our immunoblot analysis of soleus muscle revealed impaired peripheral insulin signaling via downstream AKT phosphorylation in both WD and WD + STZ mice following exogenous insulin administration. This raises questions regarding whether the glucose phenotype observed in WD + STZ mice is solely attributed to impaired endogenous insulin secretion or if it is also influenced by peripheral insulin resistance related to elevated circulating insulin levels due to WD feeding. The WD + STZ model, however, may be appropriate for studying later stages of T2D, as progressive beta cell dysfunction is associated with increasing hemoglobin A1C levels in human patients in late stages of diabetes (Fonseca, 2009). Further investigations are needed to assess the integrity of pancreatic beta cells in both groups to elucidate the mechanisms underlying glucose intolerance in this model.

Obesity and T2D are established risk factors for cardiovascular diseases via downstream consequences of chronic inflammation, poor glycemic control, and metabolic dysfunction (Powell-Wiley et al., 2021; Sudo et al., 2022). Changes in peripheral insulin signaling specifically impact neuronal function that leads to neuropathy as well as cardiac complications such as atherosclerosis, hypertension, and heart failure through mechanisms that involve lipid metabolism, inflammation, and oxidative stress (Ag et al., 2018; Powell-Wiley et al., 2021; Sudo et al., 2022). Considering our previous findings of cardiac dysfunction and pre-heart failure with preserved ejection fraction (HfpEF) in WD-fed mice, we hypothesized that STZ would exacerbate these complications (Hatahet et al., 2023). We discovered that both WD and WD + STZ mice developed early cardiac dysfunction, characterized by elevated heart rate, and decreased GLS. Notably, WD + STZ mice exhibited a higher derangement in GLS following STZ injection, which may indicate additional disease pathologies resulting from compromised insulin secretion affecting glucose homeostasis, such as diabetic cardiomyopathy and cardiac autonomic neuropathy, which remain poorly understood (Nakamura et al., 2022; Sudo et al., 2022). To further explore cardiac function, we employed HRV analysis, which demonstrated reduced HRV in both the WD and WD + STZ groups. We observed decreased protein levels of sympathetic (TH) and parasympathetic (CHAT) neuronal markers in the cardiac tissue, which, in conjunction with our HRV data, may indicate a cardiac autonomic neuropathy phenotype characterized by loss of autonomic fibers and diminished HRV during disease progression (Vinik et al., 2013; Duque et al., 2021; Sudo et al., 2022). Collectively, these findings suggest cardiac autonomic dysfunction in both groups, with the added observation of worsened GLS strain in WD + STZ mice. This raises the question if the worsened systolic function observed by GLS following STZ injection is more susceptible to change in pancreatic insulin secretion. The observed cardiac neuropathy and decreased protein levels were however similar among WD and WD + STZ groups. Further investigation is needed to determine whether this is due to a loss of cardiac innervation or because of signaling dysfunction in this model.

Neuropathy is a common complication in obesity and T2D, where dyslipidemia, inflammation, and insulin resistance are major metabolic drivers in nerve injury (Callaghan and Feldman, 2013; Stino and Smith, 2017; Callaghan et al., 2018; Kazamel et al., 2021; Bonomo et al., 2022). In our previous studies, we reported neuropathic pain symptoms and neuronal complications, such as endoplasmic reticulum stress and mitochondrial dysfunction in WD-fed mice (Gavini et al., 2018; Elshareif et al., 2022). In addition to the sensory perturbations related to neuropathy, it may also lead to complications in other physiological systems that are not well characterized in mouse models. Our nociceptive behavior data revealed the development of mechanical allodynia in both WD and WD + STZ mice, while the thermal hyperalgesia phenotype was not present at week 26, indicating thermal insensitivity at this stage of neuropathy. This pattern may indicate the later stages of small fiber neuropathy, where extensive neuronal injury leads to pain insensitivity (Hicks and Selvin, 2019). Accompanying our pain data, we found that *Atf3*, a neuronal injury marker, is upregulated in the DRG. Not only does this reflect the sensory neuropathy-related pain phenotype but may also indicate sensory disfunction in other organ systems. Given our data on cardiac dysfunction, higher levels of *Atf3* may also reflect the status of the cardiac sensory nervous system, responsible for pain perception and initiating protective mechanisms (Fukuda et al., 2015). Cardiac sensory dysfunction has been associated with silent myocardial ischemia in some diabetic patients (Ieda et al., 2006; Fukuda et al., 2015). Further studies are required to determine whether there is a loss of cardiac sensory innervation, as this model may serve as an appropriate tool for studying various early cardiac disease states.

Since the underlying pathogenesis of obesity and T2D involves disruptions in energy balance, we performed indirect calorimetry at 21 weeks (Romieu et al., 2017). To minimize the confounding effects of body mass, energy expenditure was corrected to lean mass per animal, revealing similar profiles between WD and WD + STZ mice, consistent with the higher fat mass observed, as reported by others (Guo and Hall, 2011; Müller et al., 2021). Notably, both groups exhibited difficulty in adapting energy expenditure to cold exposure compared to lean mice. Although the precise physiological factors responsible for this observation remain unclear, our data may shed light on defective activation of brown adipose tissue, given previous studies on diet-induced thermogenesis (Mercer and Trayhurn, 1987; Guo and Hall, 2011; Vijgen et al., 2011; Alcalá et al., 2017). Moreover, it has been suggested that long-term consequences may include reduced activation of brown adipose tissue due to sympathetic nerve dysfunction (Wu et al., 2014; Al et al., 2019).

Numerous studies have documented that STZ administration both in lean and HFD-fed mice decreases RER as little as 2 weeks post administration under various experimental conditions (Thackeray et al., 2011; Vitzel et al., 2013). The lower RER observed in both WD and WD + STZ mice indicates increased lipid utilization as an energy source. Given the insulin resistance phenotypes in both WD and WD + STZ mice, this observation may be correlated with the presence of insulin-resistant peripheral tissues in which fatty acids become a favored energy source (Etgen and Oldham, 2000; Townsend and Tseng, 2014; Choi et al., 2015). This also reflects the overall high-fat content of WD feeding from both groups. Interestingly, however, food intake was significantly higher in WD-fed mice, with overall greater intake compared to WD + STZ mice despite similar body weight. In addition to its impact on insulin secretion, STZ reduces circulating leptin levels, which collectively engage hypothalamic mechanisms that contribute to excess food intake (Xu et al., 2018). However, studies utilizing STZ in obese rat models have demonstrated distinct patterns in feeding behavior that have emerged depending on the nutritional content of their diet. Specifically, those on a HFD have lower food intake accompanied by a decrease in expression of hypothalamic neuropeptide Y (NPY) expression, along with an increase in corticotropin releasing hormone (CRH) (Chavez et al., 1998). Given that both the WD and WD + STZ mice display similar RER, STZ may counteract obesogenic feeding behavior via central-mediated mechanisms.

Our findings have shed light on the differences and similarities in physiological status during disease progression from prediabetes to T2D across lifespan using both WD and WD + STZ mouse models. Our results indicate that both WD and WD + STZ mouse models develop the same comorbidities, including heart and somatosensory dysfunction via autonomic innervation and sensory neuropathy phenotypes. While these are the same between models, the root causes of metabolic dysfunction vary, as WD-fed mice develop peripheral insulin resistance while WD + STZ mice possess both peripheral insulin resistance and impaired insulin secretion. These findings indicate that WD + STZ mice closely recapitulate later stages of T2D, while WD feeding alone may be more appropriate for prediabetes studies. While most of our findings reveal no significant differences comorbidities between WD and WD + STZ mice, utilization of STZ more closely recapitulates the human T2D disease state rather than genetic mouse models and long-term obesogenic feeding. This model may be useful for investigating central T2D metabolic mechanisms while WD feeding may be sufficient in studying associated complications. Further studies are necessary to study the combined effects of STZ and WD feeding to provide insight on cardiometabolic diseases. For all experiments, Fisher’s LSD was used for all *post hoc* analyses which may introduce false positive results. Further studies are necessary to validate the present study to confirm observed phenotypes. Serum metabolomic studies may provide insight on disease severity and comorbidities by quantifying metabolites involved in metabolic pathways central to the development of insulin resistance and hyperglycemia in this model. As the comorbidities share common risk factors and intertwined physiological mechanisms, this may provide insight on the systemic effects of cardiac dysfunction via inflammatory, metabolic, and adrenergic pathways involved in disease progression in this model. Investigating the long-term effects of STZ in obese mice may be instrumental in studying the complications associated with metabolic disorders and their underlying mechanisms at distinct stages of T2D throughout lifespan, thereby paving the way for therapeutic approaches aimed at improving patient outcomes and reducing the burden of disease.

## Materials and methods

### Mice

All studies were conducted in accordance with the recommendations of the Guide for the Care and Use of Laboratory Animals of the National Institutes of Health and the approval of the Loyola University Chicago Institutional Animal Care and Use Committee. All methods were conducted in accordance with the Animal Research: Reporting of *In Vivo* Experiments (ARRIVE) guidelines, with the experimenter blinded to treatments. Male C57BL/6J (#000664) mice were obtained from Jackson Laboratory (Maine, United States). Mice were housed under a 12:12 h light/dark cycle and were 7 weeks old at the beginning of the paradigm. Mice received either NC (Teklad LM-485) or WD (TD88137; 42%kcal from fat, 34% sucrose by weight, and 0.2% cholesterol) for 26 weeks.

### Body composition assessment

Body weights were measured weekly throughout the paradigm. At week 21, 24 h prior to indirect calorimetry, measurements of lean, fluid, and fat mass were performed using Minispec LF50 nuclear magnetic resonance (NMR) analyzer (Bruker Corporation, Billerica, MA).

### Glucose tolerance test

Mice were fasted in the morning (4 h) and given an i.p. injection of glucose (1 g/kg BW). Fasting glucose measurements were obtained before injection as performed previously (Gavini et al., 2018; Elshareif et al., 2022). Blood glucose levels were measured using an AlphaTrak glucometer (Fisher Scientific, United States) before and after injection at various time points.

### Von Frey mechanical sensitivity

Mice were evaluated for mechanical allodynia using von Frey filaments and acclimated in testing chambers 1h before the experiment. 6 calibrated filaments (0.16; 0.4; 1; 2; 4; 6; 8 g) (North Coast Medical, California, United States) were applied for 1 s with 6 stimulations as described previously (Gavini et al., 2018; Elshareif et al., 2022).

### Thermal nociception

Mice were assessed for thermal hyperalgesia using the Hargreaves Method Plantar Test Apparatus (IITC Life Science, California, United States). Mice were acclimated for 1 h in the testing chambers and then stimulated 6 times each for a maximum of 20 s to avoid tissue injury.

### Serum insulin quantification

Tail blood was collected after 4 h fast and 30 min post-glucose injection (1 g/kg BW) in independent experiments. Samples were spun for 10 min at 2,000 g and serum were collected. Serum insulin levels were quantified using an Ultra-Sensitive Mouse Insulin ELISA Kit (Crystal Chem, Illinois, United States) following the manufacturer’s instructions. HOMA-IR scores were calculated using the following equation: [fasting glucose (mg/dL) * fasting insulin (mU/L)]/405.

### Western blot

Proteins were extracted from frozen hearts and soleus muscle explants. Tissues were submerged in ice-cold RIPA buffer (ThermoFisher, #89900) containing protease and phosphatase inhibitors (ThermoFisher, #A32959). Tissues were powdered and homogenized using a bullet blender bead lysis kit (Next Advance). The samples were spun at 800 g for 2 min at 4°C and supernatant was collected and centrifuged at 12,000 g for 10 min at 4°C. Protein concentrations were determined using a Pierce BCA Protein Assay Kit (ThermoFisher, #23225). Proteins were separated using sodium dodecyl sulfate polyacrylamide gel electrophoresis (SDS-PAGE) on 4%–20% gradient gels (Bio-Rad, #4561094) and then transferred to polyvinylidene fluoride (PVDF) membranes (ThermoFisher, #IB24002) using the iBlot 2 transfer system (ThermoFisher). Protein expression was measured by chemiluminescence using the ChemiDoc imaging system (Bio-Rad). Protein expression was quantified using Ponceau S (ThermoFisher, #A40000279). The following primary antibodies were used: Tyrosine hydroxylase, 1:1000 (Millipore Ab152), AKT, 1:1000 (Cell Signal 9272s), pAKT, 1:1000 (Cell Signal 9271s), ChAT, (1:2000, ab178850).

### Indirect calorimetry

Mice were housed in individual cages within a TSE PhenoMaster climate chamber maintained at 24.5°C and 40% humidity. Water and food were provided *ad libitum*. Oxygen consumption, carbon dioxide production, food intake, and water intake were measured using a TSE PhenoMaster system (TSE Systems, Chesterfield, MO, United States). Calorimetry and food intake data were analyzed using the CalR web-based analysis tool (version 1.3), and energy expenditure data were normalized to lean body mass.

For cold exposure experiments, climate chamber temperature was reduced to 4°C, and indirect calorimetry measurements were performed for 4 h after which mice were removed from the cages and returned to regular housing conditions.

### Echocardiography

Echocardiography was performed throughout the paradigm using Visual Sonics Vevo 3100 system equipped with MX550D transducer (Visual Sonics). Mice were anesthetized with isoflurane and measurements were performed using short-axis M-mode scans. Mice were monitored to ensure that they recovered from anesthesia without difficulty. Measurements were used to quantify HRV parameters using Kubios HRV Scientific.

### Immunohistochemistry

Fresh hearts were kept in 10% formalin solution for 24 h at 4°C then washed and transferred to a 30% sucrose solution. Fixed samples were frozen sectioned for staining. Slides were washed with PBS and blocked for 1 h at ambient temperature in PBS containing 3%BSA and 0.3% TritonX-100. Slides were incubated with primary antibody Tyrosine hydroxylase, 1:1000 (Millipore Ab152) overnight at 4°C. The next day after washing with PBS, slides were incubated with secondary antibody for 1 h at ambient temperature. After washing, slides were co-stained with DAPI and mounted for imaging.

### Quantitative PCR

RNA extraction was performed using the Acturus Picopure RNA Isolation kit (ThermoFisher, KIT0204) following the manufacturer’s instructions. 400 ng RNA from the lumbar DRG was converted to cDNA using High-Capacity cDNA Reverse Transcription Kit (Applied Biosystems, California, United States). Quantitative PCR was performed using cDNA diluted 1:10, SYBR green (Roche, #04913850001), and 10 µM forward and reverse primers for ATF3 (F: CGA​AGA​CTG​GAG​CAA​AAT​G, R: AGG​TTA​GCA​AAA​TCC​TCA​AAT​AC) and β-actin (F: ACCTTCTAC AATGAGCTGCG, R: CTG​GAT​GGC​TAC​GTA​CAT​GC). The mRNA levels were calculated relative to β-actin as the housekeeping gene using the ΔΔCt method.

### Statistical analysis

Statistical analyses were performed using GraphPad Prism 9. All data are shown as mean ± SEM. Body weight and AKT Western blot analysis were measured using repeated measures two-way analysis of variance (ANOVA) and two-way ANOVA, respectively. All other comparisons between treatments were performed using one-way analysis of variance (ANOVA). Fisher’s LSD was used for all *post hoc* analyses. Statistical significance was set at *p* < 0.05.

## Data Availability

The original contributions presented in the study are included in the article/Supplementary Material, further inquiries can be directed to the corresponding author.
