# Supplementary material for: Comparison of western diet-induced obesity and streptozotocin mouse models: insights into energy balance, somatosensory dysfunction, and cardiac autonomic neuropathy
Source: Front Physiol. 2023 Oct 11;14:1238120. doi: 10.3389/fphys.2023.1238120 (PMC10598778; doi:10.3389/fphys.2023.1238120)
Supplement: Supplementary file 1 [file Table1.DOCX]

Supplementary Material

Comparison of Western Diet-Induced Obesity and Streptozotocin Mouse Models: Insights into Energy Balance, Somatosensory Dysfunction, and Cardiac Autonomic Neuropathy

Nadia Elshareif^1^, Emily Gornick^1^, Chaitanya K. Gavini^1,2^, Gregory Aubert^1,3^, Virginie Mansuy-Aubert^1,2*^

^1^Department of Cell and Molecular Physiology, Stritch School of Medicine, Loyola University Chicago, Maywood, IL, United States

^2^Department of Biomedical Science, University of Lausanne, Lausanne, Switzerland

^3^Division of Cardiology, Department of Internal Medicine, Stritch School of Medicine, Loyola University Chicago, Maywood, IL, United States

^*^Corresponding author. Email: Virginie.mansuy-aubert@unil.ch

**Supplementary Figure 1.** **Global longitudinal strain increases post-STZ injection compared to western diet-fed mice.** Echocardiography measurements of (A) Ejection fraction and (B) global longitudinal strain (GLS) at week 10 and week 14 of the paradigm. Statistical analyses were done using one-way ANOVA (n=6-7/group). Data are mean±SEM (**p* < 0.05).

**Supplementary Figure 2. WD and WD+STZ mice develop similar somatosensory dysfunction**. (A) von Frey behavioral test for mechanical allodynia. (B) Hargreaves behavioral test for hyperalgesia. (C) mRNA levels of ATF3 in lumbar dorsal root ganglia. Statistical analyses were done using one-way ANOVA (n=6-7/group). Data are mean±SEM (**p* < 0.05, ***p < 0.005, *****p* < 0.0001).
